# Acute Ischemic and Hemorrhagic Cerebrovascular Strokes After Cardiac Surgery: Incidence, Predictors, and Outcomes

**DOI:** 10.1155/ccrp/6645363

**Published:** 2025-04-30

**Authors:** Mohamed Laimoud, Mosleh Nazzal Alanazi, Patricia Machado, Mary Jane Maghirang, Suha Althibait, Shatha Al-Mutlaq, Munirah Alomran, Imad Bou-saad, Lamees Subhi, Reem Almutairi, Renad Nadhreen, Hamza Busaleh, Sreedevi Pillai, Saranya Sidharthan, Tareq Almazeedi, Zohair Al-Halees

**Affiliations:** ^1^Cardiovascular Critical Care Department, Prince Sultan Cardiac Center, Riyadh, Saudi Arabia; ^2^Critical Care Medicine Department, Cairo University, Cairo, Egypt; ^3^Cardiovascular Critical Care Department, King Faisal Specialist Hospital and Research Center, Riyadh, Saudi Arabia; ^4^Cardiovascular Nursing Department, King Faisal Specialist Hospital and Research Center, Riyadh, Saudi Arabia; ^5^Cardiac Surgery Department, King Faisal Specialist Hospital and Research Center, Riyadh, Saudi Arabia

**Keywords:** atrial fibrillation, cardiac surgery, cerebrovascular stroke, intracranial hemorrhage, ischemic stroke, lactate, mortality

## Abstract

**Background:** Many studies have attempted to determine the incidence, predictors, and outcomes of cerebrovascular stroke after cardiac surgery, with different, sometimes contradictory, results because of differences in population risk profiles, study design, and surgical details.

**Methods:** We retrospectively reviewed the records of all adult patients who underwent cardiac surgery between January 2018 and January 2023. Univariate, multivariable, and survival analyses were performed to identify the outcomes and predictors of ischemic and hemorrhagic strokes.

**Results:** Of the 1334 patients studied, 70 (5.2%) patients had ischemic stroke, 23 (1.7%) had intracranial hemorrhage (ICH), and 9 (0.7%) had combined ischemic and hemorrhagic strokes. The patients who developed strokes had longer cardiopulmonary bypass (CPB) time (165.5 [126, 234] versus 136 [104, 171] min, *p* < 0.001) and aortic cross-clamping time (112 [79, 163] versus 89 [75, 121.5] min, *p* < 0.001), with higher rates of intra-aortic balloon pump (IABP) use (13.3% vs. 4.4%, *p* < 0.001), veno-arterial extracorporeal membrane oxygenation use (24.8% vs. 12.37%, *p* < 0.001), and mediastinal exploration for bleeding (22.9% vs. 8.9%, *p* < 0.0011). The patients who developed strokes showed increased hospital mortality (37.1% vs. 5.6%, *p* < 0.001), new need for dialysis (29.5% vs. 10.7%, *p* < 0.001), higher rate of tracheostomy (13.3% vs. 1.2%, *p* < 0.001), and longer intensive care unit (ICU) stay (12 [7, 28] versus 3 [2, 8] days, *p* < 0.001) and post-ICU stay (16 [7, 39] versus 5 [3, 10] days, *p* < 0.001). Follow-up for 36.4 (21.67, 50.7) months revealed an insignificant mortality difference, but there was an increased risk of recurrent cerebrovascular strokes. Cox-proportional hazards regression showed an increased risk of hospital mortality after cardiac surgery in patients who developed acute ischemic stroke (HR: 5.075, 95% CI: 3.28–7.851, *p* < 0.001) and ICH (HR: 12.288, 95% CI: 7.576–19.93, *p* < 0.001). Logistic multivariable regression showed that increased age, hyperlactatemia, redo cardiotomy, history of old stroke, CPB time, and perioperative IABP use were the predictors of ischemic stroke. Young age, old ICH, hyperlactatemia, and hypoalbuminemia were the predictors of postoperative ICH. Postoperative ICH, ischemic stroke, atrial fibrillation, chronic kidney disease, blood lactate level 24 h after surgery, and increased age were the independent predictors of mortality.

**Conclusions:** Ischemic and hemorrhagic cerebrovascular strokes are serious complications that increase postoperative mortality and prolong hospitalization after cardiac surgery. Atrial fibrillation was not a significant predictor of postoperative stroke but was a predictor of hospital mortality. Careful attention should be given to maintaining hemodynamic stability and minimizing CPB time, especially in patients with a history of cerebrovascular strokes and redo cardiotomy.

## 1. Background

Neurological dysfunction is a serious complication of cardiac surgery and includes altered consciousness, seizures, cognitive dysfunction, and focal deficits [1–3]. Cerebrovascular stroke after surgery is defined as an evident ischemic or hemorrhagic brain infarction that occurs during surgery or within the first 30 days postoperatively [4]. Stroke after cardiotomy is a devastating, multifaceted complication linked to increased mortality, prolonged hospitalization, increased costs, and impaired quality of life [1, 2, 5–7]. A thorough understanding of the relation of strokes and cardiac surgery is very important to improve the patients' outcomes and reduce the healthcare burdens and costs of treatment. The improvement of percutaneous cardiac interventions increased the risk profiles of patients subjected to cardiac surgery like advanced age and the presence of advanced diabetes mellitus, renal impairment, aortic calcification, or cerebrovascular diseases. Despite the advancements and new technologies of surgical and anesthesiologic techniques with intraoperative neurological monitoring, the occurrence of ischemic and hemorrhagic strokes is still challenging to diagnose early and effectively manage.

Many studies have attempted to determine the predictors and outcomes of postoperative stroke with different, sometimes contradictory, results, owing to differences in study design, population characteristics, and surgical details [2–9]. Most postoperative strokes occur early after cardiac surgery which confirms the direct relation to surgical details like aortic-clamping, cardiopulmonary bypass (CPB), proximal aortic grafting, which will result in cerebral hypoperfusion ± atheroembolism [7]. Moreover, there is limited data regarding intracranial hemorrhage (ICH) after cardiac surgery [10, 11]. The effects of surgical details and CPB vary according to the patients' risk profiles including underlying cerebrovascular disease, age, and concomitant diseases. We reviewed a cohort of adult patients who underwent cardiac surgery to identify the incidence, outcomes, and potential predictors of ischemic and hemorrhagic strokes after cardiac surgery.

## 2. Methods

The study enrolled all patients ≥ 18 years of age who underwent cardiotomy. The patients were divided according to occurrence of postoperative brain stroke, based on computed tomography (CT) or magnetic resonance imaging (MRI), into stroke and nonstroke groups. The stroke group was further subdivided into ischemic and hemorrhagic groups. Patients with ischemic stroke complicated with hemorrhagic transformation were included in the ischemic stroke group.

Acute stroke has been defined as neurological dysfunction that persists for 24 h because of brain focal vascular occlusion or rupture [12]. It is subdivided into ischemic and hemorrhagic types according to the vascular injury. The ICH includes four subtypes according to the location of blood accumulation: intracerebral, subarachnoid, extradural, and subdural hemorrhages [13].

All studied variables were collected from the hospital electronic records, and follow-up data were collected from hospital visits or phone calls. The preoperative variables included patient variables such as age, sex, body mass index, risk factors for cardiovascular and cerebrovascular diseases, left ventricular ejection fraction, antiplatelet and anticoagulant drugs, and laboratory workup. Perioperative details included type of surgery, intra-aortic balloon pump (IABP) use, aortic cross-clamping (ACC) time, CPB time, and use of veno-arterial extracorporeal membrane oxygenation (VA-ECMO). The laboratory variables included peak blood lactate and levels 24 h after surgery. The primary outcome was hospital mortality, and secondary outcomes included the length of intensive care unit (ICU) stay and hospitalization, occurrence of acute kidney injury (AKI), and new need for dialysis. Follow-up outcomes included mortality and cerebrovascular stroke recurrence.

### 2.1. Statistical Analysis

Data were coded and tested for normality. Non-normally distributed data were summarized as medians with interquartile ranges and compared using the Wilcoxon rank-sum test. For categorical variables, data were presented as frequencies with proportions, and comparisons were performed using the chi-squared or Fisher's exact test, as appropriate. Kaplan–Meier analysis was performed to obtain the survival curves of the studied groups, and log-rank tests were performed to determine statistical significance. Cox-proportional hazard regressions were used to get the hazard ratios (HRs) and 95% confidence intervals (95% CI) for postoperative ischemic and hemorrhagic strokes. The variables that were clinically and statistically significant in the univariate analysis were included in the forward multivariable logistic regression models to obtain the odds ratios (with 95% CI) for predicting hospital mortality and cerebrovascular stroke after cardiac surgery. The models were evaluated using the Hosmer–Lemeshow test of goodness and variance inflation test. Statistical analysis was performed using the Statistical Package for the Social Sciences (SPSS) version 28 (IBM Corp., USA), and significance was determined as a two-sided *p* < 0.05.

## 3. Results

### 3.1. Characteristics of the Patients Studied

**Figure 1:**
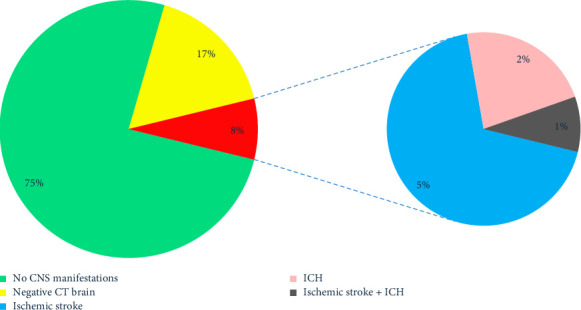
The chart shows that 328 (24.4%) patients had postoperative neurological manifestations, 223 (16.7%) had negative brain scans, and 105 (7.9%) had focal brain damage. Seventy (5.2%) patients had ischemic stroke, 23 (1.7%) had ICH, and 9 (0.7%) had combined ischemic and hemorrhagic strokes.

A total of 1334 patients were studied: 328 (24.4%) patients had postoperative neurological manifestations, 223 (16.7%) had negative brain scans, 70 (5.2%) had ischemic stroke, 23 (1.7%) had ICH, and 9 (0.7%) had combined ischemic and hemorrhagic strokes (Figure 1).

**Figure 2:**
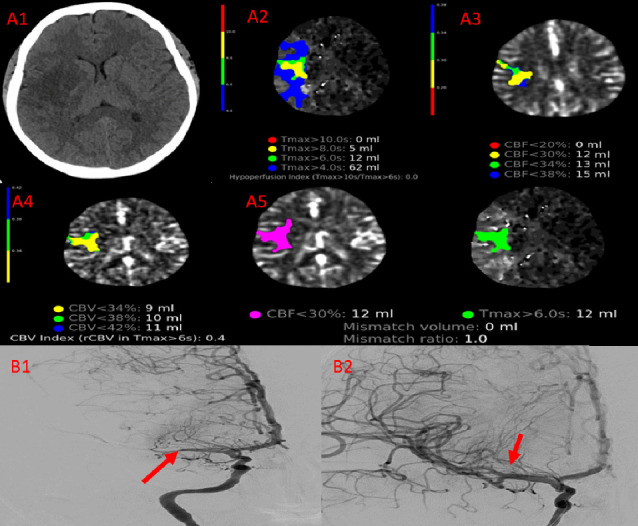
A 29-year-old female with rheumatic heart disease and chronic atrial fibrillation developed facial drooping and acute hemiparesis 24 h after redo mitral valve replacement. Computed tomography of the brain and angiography revealed acute large right middle cerebral artery (MCA) territory ischemic insult, large vessel occlusion involving the M1 segment of the right MCA, and impaired perfusion parameters (A1–5). Cerebral angiography showed total occlusion of M1 branch of the right MCA (B1). Image B2 shows the thrombolysis in cerebral infarction Flow III after thrombus aspiration, with areas of narrowing consistent with nonflow limiting vasospasm. The patient was discharged without any residual weakness.

**Figure 3:**
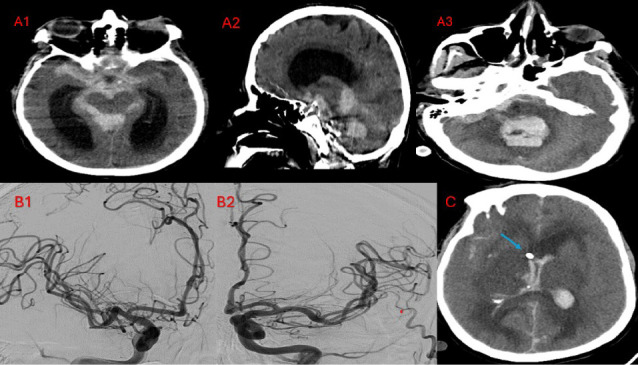
A 56-year-old man with extensive subarachnoid hemorrhage (A1) and intraventricular hemorrhage observed in the fourth ventricle (A3) associated with resultant active hydrocephalus (A1 and 2) presented with sudden loss of consciousness 36 h after third redo mitral valve replacement and tricuspid valve repair. Right (B1) and left (B2) cerebral angiograms excluded arteriovenous malformations or aneurysmal dilatation and showed severe attenuation of distal branches of the left middle cerebral artery (MCA) and posterior circulation. Image C shows the tip of the right frontal external ventricular drain (blue arrow) in the right ventricle, diffuse bilateral cerebral hemispheric sulci effacement with loss of gray-white matter differentiation, and multiple hypodensities, especially in the right MCA territory infarction (likely related to underlying vasospasm) 5 days after the loss of consciousness. The patient died after 23 days of hospital stay.

**Figure 4:**
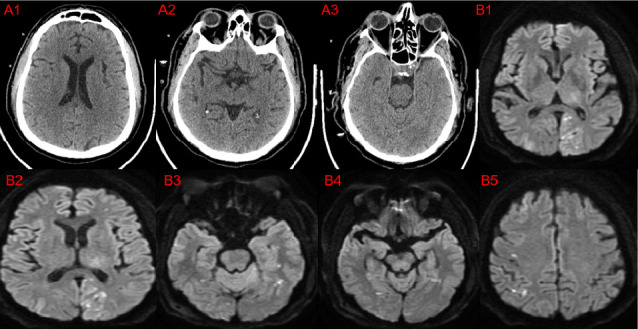
A 57-year-old man who underwent on-pump CABG and mitral valve repair with a cardiopulmonary bypass time of 221 min and aortic cross-clamping time of 191 min. Postoperative nonawakening occurred, computed tomography of the brain did not show brain vascular insult (A1–3), and CT angiography did not show large vessel occlusion. Magnetic resonance imaging of the brain (B1–5) revealed multiple patchy foci of restricted diffusion noted in the external watershed zone of both cerebral hemispheres involving bilateral frontoparietal, temporal, and occipital lobe cortices. Faint high signal areas with mild restricted diffusion are also noted in the left thalamus and both cerebellar hemispheres.

Forty-four (3.3%) patients had unilateral ischemic stroke, and 35 (2.6%) patients had bilateral ischemic infarctions. Twenty-one (1.6%) patients had unilateral ICH, and 11 (0.8%) patients had bilateral ICH. Of the 32 patients with ICH, 16 (1.2%) had subdural hematoma (SDH), 14 (1.05%) had intracerebral bleeding, 1 (0.1%) had extradural hemorrhage (EDH), and 1 (0.1%) had both SDH and EDH. The median time from surgery to stroke diagnosis was 3.9 (IQR: 2.1, 9) days (Figures 2, 3, 4).

The patients with postoperative stroke had higher preoperative rates of infective endocarditis (14.3% vs. 5.3%, *p* < 0.001), redo cardiac surgery (42.9% vs. 21.1%, *p* < 0.001), old ischemic stroke (21% vs. 5%, *p* < 0.001), and old ICH (3.8% vs. 0.7%, *p*=0.015), with significant postoperative hyperlactatemia, anemia, thrombocytopenia, and hypoalbuminemia compared with the patients without postoperative stroke (Tables 1, 2).

The patients with postoperative stroke also had longer CPB time (165.5 [126, 234] versus 136 [104, 171] min, *p* < 0.001) and ACC time (112 [79, 163] versus 89 [75, 121.5] min, *p* < 0.001) with higher rates of perioperative IABP use (13.3% vs. 4.4%, *p* < 0.001), perioperative VA-ECMO support (24.8% vs. 12.37%, *p* < 0.001), and mediastinal exploration for bleeding (22.9% vs. 8.9%, *p* < 0.001). (Table 3).

Subgroup analysis showed that patients with ischemic stroke were older with longer CPB and ACC times and higher rates of old ischemic stroke, redo cardiotomy, infective endocarditis, and perioperative IABP and VA-ECMO use than those without ischemic stroke. Patients with postoperative ICH were younger with longer CPB and ACC times and higher frequencies of old ischemic and hemorrhagic strokes, redo cardiotomy, infective endocarditis, and perioperative VA-ECMO use and lower rates of diabetes mellitus and systemic hypertension than those without postoperative ICH (Table 4).

### 3.2. Outcomes and Regression Analysis

The patients who developed cerebrovascular stroke showed increased hospital mortality (37.1% vs. 5.6%, *p* < 0.001), higher frequencies of AKI (49.5% vs. 21.7%, *p* < 0.001), new need for dialysis (29.5% vs. 10.7%, *p* < 0.001), higher rate of tracheostomy (13.3% vs. 1.2%, *p* < 0.001), and longer ICU stay (12 [7, 28] versus 3 [2, 8] days, *p* < 0.001) and post-ICU stay (16 [7, 39] versus 5 [3, 10] days, *p* < 0.001). Follow-up for 36.4 (21.67, 50.7) months showed an insignificant mortality difference, but there was an increased rate of recurrent cerebrovascular strokes (Table 5). In the subgroup analysis, patients with either ischemic or hemorrhagic stroke had increased hospital mortality, AKI, new need for dialysis, and tracheostomy with prolonged hospitalization. However, the patients who developed ICH showed increased recurrence and mortality during follow-up (Table 6).

**Figure 5:**
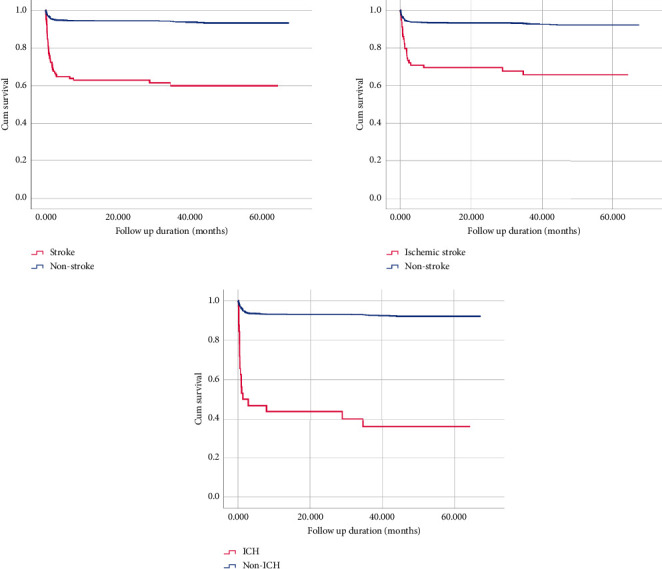
Kaplan–Meier curves showing significantly decreased survival with postoperative ischemic and hemorrhagic strokes (log-rank *p* < 0.001).

Cox-proportional hazard regression showed an increased risk of hospital mortality after cardiac surgery in patients who developed acute cerebral stroke (HR: 7.588, 95% CI: 5.186–11.103, *p* < 0.001), ischemic stroke (HR: 5.075, 95% CI: 3.28–7.851, *p* < 0.001), or ICH (HR: 12.288, 95% CI: 7.576–19.93, *p* < 0.001). Kaplan–Meier curves revealed significantly increased mortality with ischemic and hemorrhagic cerebrovascular strokes (log-rank *p* < 0.001). (Figure 5).

Logistic multivariable regression for postoperative ischemic stroke showed that increased age (odds ratio [OR]: 1.029, 95% CI: 1.01–1.048, *p*=0.003), hyperlactatemia (OR: 1.287, 95% CI: 1.183–1.431, *p* < 0.001), redo cardiotomy (OR: 2.835, 95% CI: 1.574–5.104, *p*=0.001), history of old stroke (OR: 4.683, 95% CI: 2.364–9.278, *p* < 0.001), CPB time (OR: 1.016, 95% CI: 1.003–1.34, *p*=0.042), and perioperative IABP use (OR: 2.431, 95%CI: 1.003–5.892, *p*=0.043) were the predictors. Infective endocarditis, ACC time, and perioperative VA-ECMO use were not statistically significant in the regression model. Logistic multivariable regression was performed to determine the independent predictors of ICH and revealed that young age (OR: 0.96, 95% CI: 0.94–0.99, *p*=0.006), old ICH (OR: 6.49, 95% CI: 3.266–9.354, *p* < 0.001), hyperlactatemia (OR: 1.267, 95% CI: 1.123–1.43, *p* < 0.001), and hypoalbuminemia (OR: 0.9, 95% CI: 0.832–0.973, *p*=0.009) were the predictors of postoperative ICH (Table 7).

The logistic multivariable regression revealed that postoperative ICH (OR: 6.968, 95% CI: 2.319–20.942, *p*=0.001), ischemic stroke (OR: 2.43, 95% CI: 1.06–5.57, *p*=0.036), atrial fibrillation (OR: 3.297, 95% CI: 1.687–6.443, *p* < 0.001), CKD (OR: 2.96, 95% CI: 1.484–5.896, *p*=0.002), blood lactate at 24 h after surgery (OR: 3.267, 95% CI: 2.659–4.014, *p* < 0.001), and increased age (OR: 1.028, 95% CI: 1.002–1.069, *p*=0.027) were the independent predictors of hospital mortality after cardiac surgery (Table 8).

## 4. Discussion

Our analysis of all consecutive adult patients who underwent cardiac surgery revealed that cerebral injury occurred in 24.4% of patients, ischemic stroke in 5.2%, and ICH in 1.7%. The occurrence of stroke was associated with increased mortality and many morbidities, including the length of hospitalization and associated costs of treatment. Cerebral injury has been reported to occur in 15%–66% of patients after surgery and in 40% of patients 5 years later [14, 15]. Neurological manifestations include altered consciousness, behavioral and cognitive changes, and focal deficits. There are large variations in the reported rates of stroke and neurological problems after cardiac surgery owing to differences in patient risk factors, surgical techniques, definitions of the studied variables, study design, and brain imaging protocols [2, 5, 6, 14, 16, 17].

Bucerius et al. [14] studied 16,184 patients and reported a 4.6% incidence of ischemic stroke after cardiac surgery. The incidence varied with subgroup analysis from 1.9% after aortic valve replacement to 9.7% after double or triple valve replacement. Messé et al. [16] reported clinical strokes in 17% patients after valve replacement in patients ≥ 65 years old. Salazar et al. [5] studied 5971 patients and reported ischemic stroke in 214 (3.6%) patients with watershed infarcts in 24% of them. Raffa et al. [2] studied 2121 patients and reported major ischemic stroke in 1.7% of patients based on brain CT or MRI. Mao et al. [17] conducted a systematic review of 14 studies and reported an incidence of ischemic stroke up to 7.5% after coronary artery bypass grafting (CABG).

Because CT of the brain may fail to show focal damage and limitations of cardiac MRI after surgery, strokes may be underreported in the early postoperative stage. Pérez-Vela et al. [3] studied 688 patients and reported the failure of CT to identify significant findings in 70% of patients with ischemic stroke and the beneficial role of MRI when feasible to detect strokes. Floyd et al. [18] described the silent ischemic strokes after cardiac surgery. They performed brain MRI before and after cardiac surgery in 34 patients and reported evidence of ischemic infarctions in 18% of patients, of which 67% were clinically silent without any neurological manifestations.

There are limited data regarding ICH after cardiac surgery and mostly case reports. Yuan and Guo [11] reviewed and described 182 patients with ICH after cardiac surgery from 35 reports, mostly intracerebral bleeding. Kim et al. [10] reported a 2.6% incidence rate of ICH after cardiac CABG during a 6-year follow-up with the highest incidence within the first 30 days after surgery.

Our study revealed an association between ischemic and hemorrhagic strokes and hospital mortality. Ischemic stroke and ICH were associated with an increased risk of death and were independent predictors of mortality (HR: 5.075, 95% CI: 3.28–7.851, *p* < 0.001; OR: 2.43, 95% CI: 1.06–5.57, *p*=0.036 and HR: 12.288, 95% CI: 7.576–19.93, *p* < 0.001; OR: 6.968, 95% CI: 2.319–20.942, *p*=0.001), respectively. Moreover, there were significantly higher rates of AKI, new need for dialysis, tracheostomies, length of ICU and ward stays, with increased treatment costs in patients who had ischemic and hemorrhagic strokes. Our results are consistent with those of other studies that have reported outcomes after cardiac surgeries [2, 5–7, 10]. Gaudino et al. [7] conducted a large meta-analysis including 36 studies with 174,969 adult patients and reported high mortality in patients who developed stroke, especially early “on-awakening” stroke. Early postoperative stroke that occurs with awakening or after endotracheal extubation is linked to intraoperative events and aortic manipulation, whereas delayed stroke refers to stroke that occurs after a symptom-free period and is usually related to patient risk factors, atrial fibrillation, and cerebrovascular disease [7, 19]. The intraoperative risk factors include cerebral hypoperfusion and atheroembolism with aortic clamping, insertion and removal of cannulas, and proximal aortic grafting.

Our analysis of risk factors revealed the significantly prolonged CPB and ACC times in patients with ischemic and hemorrhagic strokes, and CPB was an independent predictor of postoperative ischemic stroke (OR: 1.016, 95% CI: 1.003–1.34, *p* = 0.042). However, there are contradictory reports on the association between CPB duration and cerebrovascular insults. Many reports linked early postoperative neurological insults to CPB time and ACC [5–8, 20, 21], whereas other reports did not find this association [2, 22]. A CPB time > 120 min was identified as a risk factor for early stroke [14, 20]. A large meta-analysis of 174,969 patients confirmed an inverse association between off-pump surgery and early strokes [7]. John et al. [21] reported that patients with aortic calcification had a more than threefold risk of early stroke and recommended a safe aortic approach, including avoiding proximal anastomoses, using sequential grafts, femoral cannulation, and off-pump surgeries, if feasible. Gas embolism during CPB remains a risk factor for intraoperative stroke despite de-airing techniques, and results in significant focal or generalized brain injury [23, 24]. Carbon dioxide flooding has been proposed to decrease postoperative neurological dysfunction with conflicting results [25, 26].

A history of ischemic or hemorrhagic stroke was a significant predictor of postoperative stroke in our study. Our findings are consistent with those of previous studies that reported a link between a history of cerebrovascular disease and postoperative delayed stroke [6–8, 14, 21, 22, 27]. Atrial fibrillation was not a significant predictor of postcardiotomy stroke in our multivariable models; however, it was an independent predictor of hospital mortality (OR: 3.297, 95% CI: 1.687–6.443, *p* < 0.001) in our cohort. Most of our patients with atrial fibrillation already required therapeutic anticoagulation because of valve surgery to decrease the risk of thromboembolism. Hogue et al. [27] found no association of atrial fibrillation with postcardiotomy stroke unless it was associated with low cardiac output. Atrial fibrillation was not a predictor of postcardiotomy stroke in many reports [2, 14, 20] but it was a predictor in few reports [6, 22]. Postoperative atrial fibrillation (POAF) was linked to postoperative stroke and worse outcomes in patients who underwent isolated CABG and did not receive therapeutic anticoagulation [6, 28, 29]. POAF was associated with worse outcomes after cardiac surgery, including increased mortality [30, 31]. Perioperative prophylaxis with beta blockers or amiodarone decreases the risk of POAF. Preoperative screening for the need for ablation, left atrial appendage ligation, and posterior pericardiectomy decreased the risk of POAF that was associated with worse outcomes [32].

Blood lactate levels were used as markers of tissue hypoperfusion and microcirculation dysfunction. Hyperlactatemia was a significant variable in the multivariable analysis of the postoperative stroke models. Moreover, blood lactate level 24 h after surgery, which represents persistent tissue hypoperfusion and delayed clearance, was an independent predictor of mortality in our cohort (OR: 3.267, 95% CI: 2.659–4.014, *p* < 0.001). Hyperlactatemia and delayed clearance are linked to increased mortality and poor outcomes after cardiac surgery [33–37].

Previous cardiotomy was a significant risk factor for ischemic stroke (OR: 2.835), which is consistent with previous reports [2, 14]. Previous cardiotomy increases the risk of bleeding, hypotension, CPB duration, and aortic manipulation, with an increased risk of embolism. Hypoalbuminemia was a predictor of ICH after cardiac surgery in our cohort analysis, which could be explained by fluid shifts and cerebral volume fluctuations, especially with systemic inflammatory response (SIRS), thrombocytopenia, and anticoagulation. Hypoalbuminemia after cardiac surgery occurs because of many factors, including bleeding, fluid resuscitation, CPB circulation, and tissue injury with SIRS [38, 39]. Blood albumin has been shown to exert neuroprotective effects via anti-inflammatory, anti-apoptotic, and antioxidant effects [40]. Berbel-Franco et al. [39] studied 2818 patients and reported an association between hypoalbuminemia 24 h after surgery and worse outcomes, including mortality, AKI, sepsis, hemorrhagic complications, and ICU stay.

Cerebrovascular stroke after cardiac surgery is a multifaceted complication that affects patient outcomes, diagnosis and management challenges, rehabilitation, increased costs, and healthcare burdens. Therefore, a multidisciplinary approach should be considered before cardiac surgery. Careful assessment of patients before surgery should be performed to identify the risk factors for stroke and to choose the surgical approach with minimal risks, such as minimal aortic handling, use of CPB, or the off-pump approach. Avoiding intraoperative hemodynamic instability, minimal aortic handling, and reducing CPB and ACC times decrease the risk of postoperative stroke [19]. Cerebral oximetry allows the continuous monitoring of brain oxygenation and early detection of brain hypoxemia [41]. Early postoperative neurological examination, including pupillary reflex and any motor deficit, is crucial for the early identification of postoperative stroke and allows immediate care, including thrombectomy of the large cerebral vessels and anticoagulation management. Implementing the stroke code policy in our cardiac center allowed for the early detection of ischemic stroke and thrombectomy, which successfully restored blood flow and neurological recovery in eligible patients. Mechanical thrombectomy was reported to be beneficial after cardiac surgery if stroke was detected early and was associated with large-vessel occlusion [42, 43]. A multidisciplinary approach is crucial after cardiac surgery for patients who develop neurological dysfunction and should involve critical care, neurology, neurosurgery, physiotherapy, speech therapy, swallowing assessment, and rehabilitation teams. The aim of this approach is to deliver comprehensive assessment and immediate individualized patient care that will help minimize damage and improve outcomes and quality of life. A multidisciplinary approach for stroke after cardiac surgery has been reported to significantly increase survival [44].

## 5. Conclusions

Acute ischemic and hemorrhagic cerebrovascular strokes are serious, multifaceted complications that increase postoperative mortality and prolong hospitalization after cardiac surgery. Atrial fibrillation was not a significant predictor of postoperative stroke but was a predictor of hospital mortality. Careful attention should be given to maintaining hemodynamic stability and minimizing CPB and ACC times, especially in patients with a history of cerebrovascular stroke and redo cardiotomy.

### 5.1. Limitations

This was a retrospective, single-center study with missing intraoperative details such as cerebral oximetry. There was little data regarding preoperative ascending aorta assessment. Most of the missing data were laboratory variables during the postoperative stay. We used only data during the first 48 h after surgery, and missing data were not replaced but were managed as pairwise missed, not listwise.

## Figures and Tables

**Table 1:** Clinical characteristics of the study patients.

Variables		All patients (*n* = 1334)	Stroke group (*n* = 105, 7.9%)	Group without stroke (*n* = 1229, 92.1%)	*p* value
Age (years)		50.15 (39, 62)	55 (41, 63)	50 (39, 61.9)	0.141

Sex, male (*n*, %)		797 (59.7)	64 (61)	733 (59.6)	0.79

BMI (kg/m^2^)		27.7 (23.3, 31.8)	26.4 (22.1, 30.5)	27.8 (23.4, 31.8)	0.021

Diabetes mellitus (*n*, %)		548 (41.1)	43 (41)	505 (41.1)	0.98

Systemic hypertension (*n*, %)		783 (58.7)	54 (51.4)	729 (59.3)	0.115

Chronic kidney disease (*n*, %)		227 (17)	22 (21)	205 (16.7)	0.263

ESRD on dialysis (*n*, %)		49 (3.7)	4 (3.8)	45 (3.7)	0.79

Bronchial asthma/COPD (*n*, %)		88 (6.6)	11 (10.5)	77 (6.3)	0.09

Prior myocardial infarction (*n*, %)		145 (10.9)	12 (11.4)	133 (10.8)	0.84

Prior PCI (*n*, %)		139 (10.4)	8 (7.6)	131 (10.7)	0.328

Prior cardiac surgery (*n*, %)		304 (22.8)	45 (42.9)	259 (21.1)	< 0.001

Infective endocarditis (*n,* %)		80 (6)	15 (14.3)	65 (5.3)	< 0.001

Preoperative LV-EF (*n*, %)	> 55%	601 (45.1)	39 (37.1)	562 (45.7)	0.243
	45%–55%	348 (26.1)	30 (28.6)	318 (25.9)	
	35%–45%	136 (10.2)	10 (9.5)	126 (10.3)	
	< 35%	249 (18.7)	26 (24.8)	223 (18.1)	

Left atrium diameter (cm)		4.44 ± 0.93	4.5 ± 0.64	4.4 ± 0.37	0.103

Preoperative IABP (*n*, %)		13 (1)	4 (3.8)	9 (0.7)	0.015

Preoperative VA-ECMO (*n*, %)		18 (1.35)	4 (3.8)	19 (1.5)	0.101

Preoperative atrial fibrillation (*n*, %)		341 (25.6)	22 (21)	319 (26)	0.259

Old ischemic stroke (*n*, %)		84 (6.3)	22 (21)	62 (5)	< 0.001

Old ICH (*n*, %)		13 (1)	4 (3.8)	9 (0.7)	0.015

Epilepsy (*n*, %)		25 (1.9)	3 (2.9)	22 (1.8)	0.44

History of TIAs (*n*, %)		34 (2.5)	5 (4.8)	29 (2.4)	0.182

CAS ≥ 70% (*n*, %)		19 (0.014)	1 (1)	18 (1.5)	0.54

CAS 50%–69% (*n*, %)		15 (0.011)	2 (1.9)	13 (1.1)	0.34

Hypothyroidism (*n*, %)		96 (7.2)	10 (9.5)	86 (7)	0.336

Autoimmune disease (*n*, %)		156 (11.7)	14 (13.3)	142 (11.6)	0.35

Peripheral vascular disease (*n*, %)		35 (2.6)	6 (5.7)	29 (2.4)	0.051

Preoperative drugs (*n*, %)	Aspirin	516 (38.7)	32 (30.5)	484 (39.4)	0.072
	Clopidogrel	147 (11)	5 (4.8)	142 (11.6)	0.033
	Ticagrelor	17 (1.3)	0	17 (1.4)	0.39
	Warfarin	360 (27)	29 (27.6)	331 (26.9)	0.879
	Rivaroxaban	37 (2.8)	1 (1)	36 (2.9)	0.356
	Apixaban	49 (3.7)	4 (3.8)	45 (3.7)	0.791

Abbreviations: BMI, body mass index; CAS, carotid artery stenosis; COPD, chronic obstructive pulmonary disease; ESRD, end-stage renal disease; IABP, intra-aortic balloon pump; ICH, intracranial hemorrhage; LV-EF, left ventricular ejection fraction; PCI, percutaneous coronary intervention; TIAs, transient ischemic attacks; VA-ECMO, veno-arterial extracorporeal membrane oxygenation.

**Table 2:** Laboratory variables of the study patients.

Variables	All patients (*n* = 1334)	Stroke group	Group without stroke	*p* value
Preoperative variables				
Hemoglobin (gm/L)	119 (104, 137)	108 (99, 127)	121 (105,137)	< 0.001
Hematocrit (%)	38 (33, 42)	34 (31, 39)	39 (33, 42)	< 0.001
Platelet count (10^9^/L)	228.5 (170, 278)	223 (160, 284)	229 (171, 278)	0.513
White blood cells (10^9^/L)	7.54 (5.68, 10.36)	8.39 (6.06, 11.7)	7.48 (5.66, 10.16)	0.043
INR	1.2 (1.1, 1.4)	1.3 (1.1, 1.6)	1.2 (1.1, 1.4)	0.043
Fibrinogen (g/L)	2.95 (2.39, 3.51)	2.78 (2.22, 3.29)	2.96 (2.4, 3.56)	0.132
Serum creatinine (umol/L)	80 (65, 104)	85 (67,107)	80 (65,103)	0.221
Serum bilirubin (umol/L)	10.7 (6.4, 19)	13 (7, 24)	10.3 (6.3, 19)	0.025
Blood urea (mmol/L)	5.9 (4.5, 8.8)	6.3 (4.9, 9.6)	5.8 (4.5, 8.8)	0.182
Serum Na (mmol/L)	140 (137, 142)	140 (135, 143)	140 (138, 142)	0.619
Albumin (gm/L)	39.2 (35, 42.6)	36.3 (31, 39.2)	39.5 (35.6, 42.8)	< 0.001
Postoperative variables				
Peak blood lactate (mmol/L)	6.8 (5.4, 9.4)	11.4 (8.2, 15.7)	6.4 (5.3, 8.6)	< 0.001
Lactate at 24 h (mmol/L)	1.7 (1.3, 2.4)	3.1 (1.9, 4.7)	1.6 (1.3, 2.3)	< 0.001
Hemoglobin-D1 (gm/L)	97 (87, 107)	93 (84.5, 103)	97 (87, 107)	0.015
Hematocrit-D1 (%)	30 (27, 32)	28 (26, 31)	30 (27, 32)	0.003
Hemoglobin-D2 (gm/L)	92 (85, 101)	88 (79, 100)	92 (85, 101)	< 0.001
Platelet count-D1 (10^9^/L)	148 (105.5, 197)	124 (80, 170)	151 (108, 198)	< 0.001
Platelet count-D2 (10^9^/L)	134 (97, 171)	113 (77, 152)	136 (101, 173)	< 0.001
aPTT-D1	38.2 (35.1, 42.9)	42.55 (36.5, 54)	38 (35, 41)	< 0.001
aPTT-D2	42 (38.2, 52)	42 (38.6, 47.5)	42 (38.2, 52.3)	0.95
Fibrinogen-D1 (g/L)	3 (2.5, 3.66)	2.7 (2.23, 3.29)	3.01 (2.52, 3.69)	0.001
Fibrinogen-D2 (g/L)	3.79 (2.88, 4.84)	3 (2.45, 4.01)	3.88 (2.93, 4.92)	< 0.001
Serum creatinine-D1 (umol/L)	83 (67, 114)	88 (74, 124)	83 (67, 114)	0.105
Serum bilirubin-D1 (umol/L)	18.6 (10.9, 29.7)	24.25 (13.9, 37.5)	18 (10.7, 28.5)	< 0.001
Blood urea-D1 (mmol/L)	6.3 (4.9, 9.4)	7.2 (5.15, 9.85)	6.2 (4.8, 9.4)	0.068
Blood urea-D2 (mmol/L)	7.6 (5.3, 12)	8.2 (6.7, 11.8)	7.5 (5.2, 12)	0.033
Albumin-D1 (gm/L)	37 (34.1, 40.6)	34.45 (30.7, 38)	37 (34.3, 40.7)	< 0.001
Albumin-D2 (gm/L)	37.3 (34.5, 40.2)	35 (31.8, 38)	37.6 (34.8, 40.4)	< 0.001

*Note:* Data are presented as median with the 25th and 75th quartiles.

Abbreviations: APTT, activated partial thromboplastin time; INR, international normalized ratio.

**Table 3:** Perioperative details of the study patients.

Variables		All patients (*n* = 1334)	Stroke group (*n* = 105, 7.9%)	Group without stroke (*n* = 1229, 92.1%)	*p* value
Type of surgery (*n*, %)	Isolated CABG	364 (27.3)	19 (18.1)	345 (28.1)	< 0.001
	Valve surgery	707 (52.9)	43 (40.9)	664 (54.02)	
	CABG + valve	71 (5.3)	12 (11.4)	59 (4.8)	
	Bentall's operation	32 (2.4)	9 (8.6)	23 (1.9)	
	Heart transplantation	68 (5.1)	7 (6.7)	61 (5)	
	LVAD implantation	56 (4.2)	7 (6.7)	49 (4)	
	ACHD	17 (1.3)	4 (3.8)	13 (1.1)	
	Pulmonary endarterectomy	11 (0.82)	0	11 (0.89)	
	Resection of cardiac tumor	8 (0.6)	0	8 (0.7)	

Valve surgery (*n*, %)	MVR	309 (23.16)	19 (18.1)	290 (23.6)	0.08
	AVR	188 (14.1)	18 (17.1)	171 (13.9)	
	MVR + AVR	73 (5.47)	10 (9.5)	63 (5.1)	
	TVR	19 (1.42)	0	19 (1.5)	
	MVR + TV repair	141 (10.57)	9 (8.6)	132 (10.7)	
	MVR + AVR + TV repair	59 (4.42)	7 (6.7)	52 (4.2)	
	Mitral repair	21 (1.6)	5 (4.8)	16 (1.3)	

Cardiopulmonary bypass time (min)		136 (105, 174)	165.5 (126, 234)	136 (104, 171)	< 0.001

Aortic cross-clamping time (min)		90 (75, 123)	112 (79, 163)	89 (75, 121.5)	< 0.001

Circulatory arrest (*n*, *%*)		22 (1.65)	10 (9.5)	12 (1)	< 0.001

Perioperative IABP (*n*, %)		68 (5.1)	14 (13.3)	54 (4.4)	< 0.001

Perioperative VA-ECMO (*n*, %)		178 (13.34)	26 (24.8)	152 (12.37)	< 0.001

ECMO cannulation (*n*, %)	Central	87 (6.52)	16 (15.2)	71 (5.78)	0.003
	Peripheral	81 (6.07)	7 (6.7)	74 (6.02)	
	Central then peripheral	6 (0.45)	2 (1.9)	4 (0.33)	
	Peripheral then central	4 (0.29)	1 (0.95)	3 (0.24)	

Exploration for thoracic bleeding (*n*, %)		133 (10)	24 (22.9)	109 (8.9)	< 0.001

New-onset POAF (*n*, %)		95 (7.1)	9 (8.6)	86 (7)	0.54

Atrial fibrillation (preoperative and POAF)		424 (31.8)	31 (29.5)	393 (32)	0.604

Abbreviations: AVR, aortic valve replacement; CABG, coronary artery bypass grafting; IABP, intra-aortic balloon pump; MVR, mitral valve replacement; POAF, postoperative atrial fibrillation; TV, tricuspid valve; TVR, tricuspid valve replacement; VA-ECMO, veno-arterial extracorporeal membrane oxygenation.

**Table 4:** Clinical and laboratory characteristics of the ischemic and hemorrhagic strokes.

Variables	Ischemic stroke group (*n* = 79, 5.9)	Nonischemic stroke group (*n* = 1255, 94.1%)	*p* value	Hemorrhagic stroke group (*n* = 32, 2.4%)	Nonhemorrhagic stroke group (*n* = 1302, 97.6%)	*p* value
Age (years)	57 (47.5, 64)	50 (39, 61.9)	0.009	41.85 (23, 55.5)	51 (40, 62)	0.014
Sex, male (*n*, %)	52 (65.8)	745 (59.4)	0.26	14 (43.8)	783 (60.1)	0.062
BMI (kg/m^2^)	26.6 (22.6, 30.5)	27.75 (23.3, 31.8)	0.07	26.35 (20.2, 30.75)	27.7 (23.4, 31.8)	0.12
Diabetes mellitus (*n*, %)	38 (48.1)	510 (40.6)	0.19	5 (15.6)	543 (41.7)	0.003
Systemic hypertension (*n*, %)	44 (55.7)	739 (58.9)	0.58	11 (34.4)	772 (59.3)	0.005
CKD (*n*, %)	15 (19)	212 (16.9)	0.63	7 (21.9)	220 (16.9)	0.46
ESRD on dialysis (*n*, %)	4 (5.1)	45 (3.6)	0.53	0	49 (3.8)	0.63
Bronchial asthma/COPD (*n*, %)	7 (8.9)	81 (6.5)	0.4	3 (9.4)	85 (6.5)	0.47
Autoimmune disease (*n*, %)	16 (20.3)	204 (16.3)	0.35	11 (34.4)	209 (16.1)	0.006
Old cerebrovascular stroke (*n*, %)	19 (24.1)	65 (5.2)	< 0.001	7 (21.9)	77 (5.9)	0.003
Old ICH (*n*, %)	2 (2.5)	11 (0.9)	0.18	3 (9.4)	10 (0.8)	0.003
Prior cardiac surgery (*n*, %)	33 (41.8)	271 (21.6)	< 0.001	15 (46.9)	289 (22.2)	0.001
Infective endocarditis (*n*, %)	10 (12.7)	70 (5.6)	0.023	5 (15.6)	75 (5.8)	0.038
Preoperative AF (*n*, %)	15 (19)	326 (26)	0.17	7 (21.9)	334 (25.7)	0.63
Perioperative IABP (*n*, %)	13 (16.5)	55 (4.4)	< 0.001	4 (12.5)	64 (4.9)	0.08
Perioperative VA-ECMO (*n*, %)	19 (24.1)	159 (12.67)	0.006	12 (37.5)	166 (12.75)	< 0.001
CPB time (min)	164 (122, 215)	136 (105, 171)	< 0.001	189 (134.5, 282.5)	136 (105, 172)	< 0.001
ACC time (min)	102 (78, 163)	90 (75, 122)	0.023	129 (98.5, 179)	90 (75, 123)	< 0.001
Circulatory arrest (*n*, %)	7 (8.9)	15 (1.2)	< 0.001	3 (9.4)	19 (1.5)	0.014
Preoperative hemoglobin (gm/L)	110 (100, 129)	120 (105, 137)	0.014	106 (99, 125.5)	120 (105, 137)	0.022
Preoperative platelet count (10^9^/L)	225 (171, 277)	229 (170, 278)	0.87	213 (104, 311)	229 (171, 278)	0.6
Peak lactate (mmol/L)	11 (7.8, 15.3)	6.5 (5.3, 8.9)	< 0.001	14.5 (10, 16.8)	6.7 (5.3, 9.2)	< 0.001
Lactate at 24 h (mmol/L)	2.8 (1.9, 4.5)	1.7 (1.3, 2.4)	< 0.001	3.8 (2.35, 5.8)	1.7 (1.3, 2.4)	< 0.001
Hemoglobin-D1 (gm/L)	94 (84, 104)	97 (87, 107)	0.054	91 (84, 104)	97 (87, 107)	0.12
Platelet count-D1 (10^9^/L)	136 (94, 179)	150 (106, 197)	0.023	100 (60, 151)	150 (107, 197)	< 0.001
Platelet count-D2 (10^9^/L)	121 (84, 153)	135 (98, 172)	0.02	92 (61, 137.5)	135 (98, 172)	0.002
Fibrinogen-D1 (g/L)	2.57 (2.22, 3.24)	3.01 (2.52, 3.67)	0.001	2.7 (2.4, 3.12)	3 (2.5, 3.67)	0.043
Fibrinogen-D2 (g/L)	3 (2.45, 4.2)	3.84 (2.9, 4.9)	0.001	3 (2.55, 3.6)	3.83 (2.89, 4.89)	0.001
Albumin-D1 (gm/L)	34.45 (31, 38.1)	37 (34.3, 40.7)	< 0.001	32.4 (29.4, 36.4)	37 (34.2, 40.6)	< 0.001
Albumin-D2 (gm/L)	35 (31.8, 38.5)	37.55 (34.8, 40.3)	< 0.001	35 (31.4, 37.4)	37.4 (34.6, 40.3)	0.001

Abbreviations: ACC, aortic cross-clamping; AF, atrial fibrillation; BMI, body mass index; CKD, chronic kidney disease; CPB, cardiopulmonary bypass; COPD, chronic obstructive pulmonary disease; ESRD, end-stage renal disease; IABP, intra-aortic balloon pump; ICH, intracranial hemorrhage; VA-ECMO, veno-arterial extracorporeal membrane oxygenation.

**Table 5:** Outcomes of the study patients.

Variables	All patients (*n* = 1334)	Stroke group (*n* = 105, 7.9%)	Group without stroke (*n* = 1229, 92.1%)	*p* value
Acute kidney injury (*n*, %)	319 (23.9)	52 (49.5)	267 (21.7)	< 0.001
New need for dialysis (*n*, %)	163 (12.2)	31 (29.5)	132 (10.7)	< 0.001
ICU days	4 (2, 9)	12 (7, 28)	3 (2, 8)	< 0.001
ECMO days	7 (5, 9)	11 (5, 15)	6 (5, 8)	0.012
Post-ICU ward days	5 (4, 10)	16 (7, 39)	5 (3, 10)	< 0.001
Tracheostomy (*n*, %)	29 (2.2)	14 (13.3)	15 (1.2)	< 0.001
Hospital mortality (*n*, %)	108 (8.1)	39 (37.1)	69 (5.6)	< 0.001
Mortality during follow-up (*n*, %)	9 (0.7)	2 (1.9)	7 (0.6)	0.154
Ischemic stroke during follow-up (*n*, %)	14 (1.1)	5 (4.8)	9 (0.7)	0.003
ICH during follow-up (*n*, %)	3 (0.2)	2 (1.9)	1 (0.1)	0.017

Abbreviations: ECMO, extracorporeal membrane oxygenation; ICH, intracranial hemorrhage; ICU, intensive care unit.

**Table 6:** Outcomes of the patients with ischemic and hemorrhagic strokes.

Variables	Ischemic stroke group (*n* = 79, 5.9)	Nonischemic stroke group (*n* = 1255, 94.1%)	*p* value	Hemorrhagic stroke group (*n* = 32, 2.4%)	Nonhemorrhagic stroke group (*n* = 1302, 97.6%)	*p* value
Acute kidney injury (*n*, %)	36 (45.6)	283 (22.5)	< 0.001	20 (62.5)	299 (23)	< 0.001
New need for dialysis (*n*, %)	18 (22.8)	145 (11.6)	0.003	17 (53.1)	146 (11.2)	< 0.001
Exploration for thoracic bleeding (*n*, %)	18 (22.8)	115 (9.2)	< 0.001	9 (28.1)	124 (9.5)	0.003
ICU days	14 (6, 38)	3 (2, 8)	< 0.001	12 (6, 28.5)	4 (2, 9)	< 0.001
ECMO days	11 (8, 15)	6 (5, 8)	0.008	11 (4, 17)	7 (5, 8)	0.57
Post-ICU ward days	16.5 (7, 36.5)	5 (3.5, 10)	< 0.001	17 (7, 61.5)	5 (4, 10)	< 0.001
Tracheostomy (*n*, %)	12 (15.2)	17 (1.4)	< 0.001	4 (12.5)	25 (1.9)	0.004
Hospital mortality (*n*, %)	24 (30.4)	84 (6.7)	< 0.001	18 (56.3)	90 (6.9)	< 0.001
Mortality during follow-up (*n*, %)	2 (2.5)	7 (0.6)	0.09	2 (6.3)	7 (0.5)	0.018
Ischemic stroke during follow-up (*n*, %)	3 (3.8)	11 (0.9)	0.045	3 (9.4)	11 (0.8)	0.004
ICH during follow-up (*n*, %)	0	3 (0.2)	1	2 (6.3)	1 (0.1)	0.002

Abbreviations: ECMO, extracorporeal membrane oxygenation; ICH, intracranial hemorrhage; ICU, intensive care unit.

**Table 7:** Multivariable logistic regression analysis of cerebrovascular strokes.

Variables	Odds ratio (OR)	Confidence interval (CI)	*p* value
Ischemic stroke			
Age	1.029	1.010–1.048	0.003
Old ischemic stroke	4.683	2.364–9.278	< 0.001
Previous cardiotomy	2.835	1.574–5.104	0.001
Infective endocarditis	1.28	0.51–3.17	0.29
Lactate peak	1.287	1.183–1.431	< 0.001
Lactate at 24 h	1.005	0.904–1.117	0.928
Perioperative IABP	2.431	1.003–5.892	0.043
Perioperative VA-ECMO	0.732	0.322–1.67	0.457
CPB time	1.016	1.003–1.34	0.042
ACC time	0.992	0.982–1.002	0.134
Exploration for bleeding	0.591	0.267–1.307	0.19
Serum albumin	0.966	0.918–1.015	0.17
Intracerebral bleeding			
Age	0.963	0.938–0.989	0.006
Old cerebral bleeding	6.49	3.266–9.354	< 0.001
Previous cardiotomy	1.423	0.598–3.384	0.425
Infective endocarditis	0.847	0.258–2.78	0.78
Lactate peak	1.267	1.123–1.43	< 0.001
Lactate at 24 h	0.869	0.737–1.024	0.09
Perioperative VA-ECMO	0.584	0.209–1.629	0.304
CPB time	1.002	0.991–1.012	0.758
ACC time	1.002	0.987–1.017	0.790
Serum albumin	0.9	0.832–0.973	0.009
Postoperative platelet count	0.967	0.943–1.005	0.435
Postoperative fibrinogen level	0.98	0.844–2.053	0.225

Abbreviations: ACC, aortic cross-clamping; CPB, cardiopulmonary bypass; IABP, intra-aortic balloon pump; VA-ECMO, veno-arterial extracorporeal membrane oxygenation.

**Table 8:** Multivariable logistic regression analysis of hospital mortality.

Variables	Odds ratio (OR)	Confidence interval (CI)	*p* value
Age	1.028	1.002–1.069	0.027
CKD	2.96	1.484–5.896	0.002
Previous cardiotomy	1.063	0.532–2.125	0.86
Infective endocarditis	0.937	0.316–2.779	0.907
Old cerebrovascular stroke	2.096	0.793–5.539	0.136
Acute ischemic stroke	2.43	1.06–5.57	0.036
ICH	6.968	2.319–20.942	0.001
Lactate at 24 h	3.267	2.659–4.014	< 0.001
Atrial fibrillation	3.297	1.687–6.443	< 0.001
Perioperative VA-ECMO	3.343	0.946–11.811	0.061

Abbreviations: CKD, chronic kidney disease; ICH, intracranial hemorrhage; VA-ECMO, veno-arterial extracorporeal membrane oxygenation.

## Data Availability

The data are available from the corresponding author.

## Nomenclature

ACC: Aortic cross-clamping
AKI: Acute kidney injury
CABG: Coronary artery bypass grafting
CKD: Chronic kidney disease
CPB: Cardiopulmonary bypass
IABP: Intra-aortic balloon pump
ICH: Intracranial hemorrhage
VA-ECMO: Veno-arterial extracorporeal membrane oxygenation

## References

[1] Wolman R. L., Nussmeier N. A., Aggarwal A. (1999). Cerebral Injury After Cardiac Surgery: Identification of a Group at Extraordinary Risk. Multicenter Study of Perioperative Ischemia Research Group (McSPI) and the Ischemia Research Education Foundation (IREF) Investigators. *Stroke*.

[2] Raffa G. M., Agnello F., Occhipinti G. (2019). Neurological Complications After Cardiac Surgery: A Retrospective Case-Control Study of Risk Factors and Outcome. *Journal of Cardiothoracic Surgery*.

[3] Pérez-Vela J. L., Ramos-González A., López-Almodóvar L. F. (2005). Complicaciones Neurológicas En El Postoperatorio Inmediato De La Cirugía Cardíaca. Aportación De La Resonancia Magnética Cerebral Neurologic Complications in the Immediate Postoperative Period After Cardiac Surgery. Role of Brain Magnetic Resonance Imaging. *Revista Espanola de Cardiologia*.

[4] Mashour G. A., Moore L. E., Lele A. V., Robicsek S. A., Gelb A. W. (2014). Perioperative Care of Patients at High Risk for Stroke During or After Non-Cardiac, Non-Neurologic Surgery: Consensus Statement From the Society for Neuroscience in Anesthesiology and Critical Care. *Journal of Neurosurgical Anesthesiology*.

[5] Salazar J. D., Wityk R. J., Grega M. A. (2001). Stroke After Cardiac Surgery: Short- and Long-Term Outcomes. *The Annals of Thoracic Surgery*.

[6] Laimoud M., Maghirang M., Alanazi M. (2022). Predictors and Clinical Outcomes of Post-coronary Artery Bypass Grafting Cerebrovascular Strokes. *The Egyptian Heart Journal*.

[7] Gaudino M., Rahouma M., Di Mauro M. (2019). Early Versus Delayed Stroke After Cardiac Surgery: A Systematic Review and Meta-Analysis. *Journal of the American Heart Association*.

[8] Naito S., Demal T. J., Sill B. (2022). Neurological Complications in High-Risk Patients Undergoing Coronary Artery Bypass Surgery. *The Annals of Thoracic Surgery*.

[9] LaPar D. J., Quader M., Rich J. B. (2015). Institutional Variation in Mortality After Stroke After Cardiac Surgery: An Opportunity for Improvement. *The Annals of Thoracic Surgery*.

[10] Kim J. H., Lee P. H., Kim H. J. (2022). Incidence and Predictors of Intracranial Bleeding After Coronary Artery Bypass Graft Surgery. *Frontiers in Cardiovascular Medicine*.

[11] Yuan S.-M., Guo J.-Q. (2001). Subsequent Intracranial Haemorrhage Following Open Heart Surgery. *Annals of The College of Surgeons Hong Kong*.

[12] Sacco R. L., Kasner S. E., Broderick J. P. (2013). An Updated Definition of Stroke for the 21st Century: A Statement for Healthcare Professionals From the American Heart Association/American Stroke Association. *Stroke*.

[13] Tenny S., Thorell W. (2025). Intracranial Hemorrhage.

[14] Bucerius J., Gummert J. F., Borger M. A. (2003). Stroke After Cardiac Surgery: A Risk Factor Analysis of 16, 184 Consecutive Adult Patients. *The Annals of Thoracic Surgery*.

[15] Mahanna E. P., Blumenthal J. A., White W. D. (1996). Defining Neuropsychological Dysfunction After Coronary Artery Bypass Grafting. *The Annals of Thoracic Surgery*.

[16] Messé S. R., Acker M. A., Kasner S. E. (2014). Stroke After Aortic Valve Surgery: Results From a Prospective Cohort. *Circulation*.

[17] Mao Z., Zhong X., Yin J., Zhao Z., Hu X., Hackett M. L. (2015). Predictors Associated With Stroke After Coronary Artery Bypass Grafting: A Systematic Review. *Journal of the Neurological Sciences*.

[18] Floyd T. F., Shah P. N., Price C. C. (2006). Clinically Silent Cerebral Ischemic Events After Cardiac Surgery: Their Incidence, Regional Vascular Occurrence, and Procedural Dependence. *The Annals of Thoracic Surgery*.

[19] Ferrante M. S., Pisano C., Van Rothem J., Ruvolo G., Abouliatim I. (2023). Cerebrovascular Events After Cardiovascular Surgery: Diagnosis, Management and Prevention Strategies. *Polish Journal of Cardio-Thoracic Surgery*.

[20] Alwaqfi N., AlBarakat M. M., Qariouti H., Ibrahim K., Alzoubi N. (2024). Stroke after Heart Valve Surgery: A Single Center Institution Report. *Journal of Cardiothoracic Surgery*.

[21] John R., Choudhri A. F., Weinberg A. D. (2000). Multicenter Review of Preoperative Risk Factors for Stroke After Coronary Artery Bypass Grafting. *The Annals of Thoracic Surgery*.

[22] Asta L., Falco D., Benedetto U. (2024). Stroke After Cardiac Surgery: A Risk Factor Analysis of 580,117 Patients From UK National Adult Cardiac Surgical Audit Cohort. *Journal of Personalized Medicine*.

[23] Orihashi K., Ueda T. (2019). De-Airing in Open Heart Surgery: Report From the CVSAP Nation-Wide Survey and Literature Review. *Gen Thorac Cardiovasc Surg*.

[24] Lou S., Ji B., Liu J., Yu K., Long C. (2011). Generation, Detection and Prevention of Gaseous Microemboli During Cardiopulmonary Bypass Procedure. *The International Journal of Artificial Organs*.

[25] Nyman J., Svenarud P., van der Linden J. (2019). Carbon Dioxide De-Airing in Minimal Invasive Cardiac Surgery, A New Effective Device. *Journal of Cardiothoracic Surgery*.

[26] Benedetto U., Caputo M., Guida G. (2017). Carbon Dioxide Insufflation During Cardiac Surgery: A Meta-Analysis of Randomized Controlled Trials. *Seminars in Thoracic and Cardiovascular Surgery*.

[27] Hogue C. W., Murphy S. F., Schechtman K. B., Dávila-Román V. G. (1999). Risk Factors for Early or Delayed Stroke After Cardiac Surgery. *Circulation*.

[28] Lahtinen J., Biancari F., Salmela E. (2004). Postoperative Atrial Fibrillation is a Major Cause of Stroke After On-Pump Coronary Artery Bypass Surgery. *The Annals of Thoracic Surgery*.

[29] Megens M. R., Churilov L., Thijs V. (2017). New-Onset Atrial Fibrillation After Coronary Artery Bypass Graft and Long-Term Risk of Stroke: A Meta-Analysis. *Journal of the American Heart Association*.

[30] Segar M. W., Marzec A., Razavi M. (2023). Incidence, Risk Score Performance, and In-Hospital Outcomes of Postoperative Atrial Fibrillation After Cardiac Surgery. *Texas Heart Institute Journal*.

[31] Steinberg B. A., Zhao Y., He X. (2014). Management of Postoperative Atrial Fibrillation and Subsequent Outcomes in Contemporary Patients Undergoing Cardiac Surgery: Insights From the Society of Thoracic Surgeons CAPS-Care Atrial Fibrillation Registry. *Clinical Cardiology*.

[32] Suero O. R., Ali A. K., Barron L. R., Segar M. W., Moon M. R., Chatterjee S. (2024). Postoperative Atrial Fibrillation (POAF) After Cardiac Surgery: Clinical Practice Review. *Journal of Thoracic Disease*.

[33] Minton J., Sidebotham D. A. (2017). Hyperlactatemia and Cardiac Surgery. *Journal of Extra-Corporeal Technology*.

[34] Mak N. T., Iqbal S., De Varennes B., Khwaja K. (2016). Outcomes of Post-Cardiac Surgery Patients With Persistent Hyperlactatemia in the Intensive Care Unit: A Matched Cohort Study. *Journal of Cardiothoracic Surgery*.

[35] Hajjar L. A., Almeida J. P., Fukushima J. T. (2013). High Lactate Levels are Predictors of Major Complications After Cardiac Surgery. *The Journal of Thoracic and Cardiovascular Surgery*.

[36] Laimoud M., Machado P., Lo M. G., Maghirang M. J., Hakami E., Qureshi R. (2024). The Absolute Lactate Levels Versus Clearance for Prognostication of Post-Cardiotomy Patients on Veno-Arterial ECMO. *ESC Heart Failure*.

[37] Biancari F., Kaserer A., Perrotti A. (2024). Hyperlactatemia and Poor Outcome After Postcardiotomy Veno-Arterial Extracorporeal Membrane Oxygenation: An Individual Patient Data Meta-Analysis. *Perfusion*.

[38] Lee E. H., Chin J. H., Choi D. K. (2011). Postoperative Hypoalbuminemia is Associated With Outcome in Patients Undergoing Off-Pump Coronary Artery Bypass Graft Surgery. *Journal of Cardiothoracic and Vascular Anesthesia*.

[39] Berbel-Franco D., Lopez-Delgado J. C., Putzu A. (2020). The Influence of Postoperative Albumin Levels on the Outcome of Cardiac Surgery. *Journal of Cardiothoracic Surgery*.

[40] Cao Y., Yao X. (2024). Acute Albumin Administration as Therapy for Intracerebral Hemorrhage: A Literature Review. *Heliyon*.

[41] Gaudino M., Benesch C., Bakaeen F. (2020). Considerations for Reduction of Risk of Perioperative Stroke in Adult Patients Undergoing Cardiac and Thoracic Aortic Operations: A Scientific Statement From the American Heart Association. *Circulation*.

[42] Jazayeri S. B., Al-Janabi O. M., Ghozy S., Rabinstein A. A., Kadirvel R., Kallmes D. F. (2024). Outcomes of Mechanical Thrombectomy for Acute Ischemic Stroke Following Cardiac Interventions: A Systematic Review and Meta-Analysis. *CardioVascular and Interventional Radiology*.

[43] D’Anna L., Abu-Rumeileh S., Merlino G. (2024). Safety and Outcomes of Mechanical Thrombectomy in Acute Ischemic Stroke Attributable to Cardiological Diseases: A Scoping Review. *Journal of the American Heart Association*.

[44] Harky A., Chow V. J., Voller C. (2024). Stroke Outcomes Following Cardiac and Aortic Surgery are Improved by the Involvement of a Stroke Team. *European Journal of Clinical Investigation*.

